# GnRH-mediated olfactory and visual inputs promote mating-like behaviors in male zebrafish

**DOI:** 10.1371/journal.pone.0174143

**Published:** 2017-03-22

**Authors:** Lei Li, Jennifer L. Wojtowicz, John H. Malin, Tao Huang, Eric B. Lee, Zijiang Chen

**Affiliations:** 1 Department of Biological Sciences, University of Notre Dame, Notre Dame, IN, United States of America; 2 Center for Reproductive Medicine, Shandong University, Jinan,China; Duke University, UNITED STATES

## Abstract

The engagement of sexual behaviors is regulated by a number of factors which include gene expression, hormone circulation, and multi-sensory information integration. In zebrafish, when a male and a female are placed in the same container, they show mating-like behaviors regardless of whether they are kept together or separated by a net. No mating-like behaviors are observed when same-sex animals are put together. Through the olfacto-visual centrifugal pathway, activation of the terminalis nerve in the olfactory bulb increases GnRH signaling in the brain and triggers mating-like behaviors between males. In zebrafish mutants or wild-type fish in which the olfacto-visual centrifugal pathway is impaired or chemically ablated, in response to odor stimulation the mating-like behaviors between males are no longer evident. Together, the data suggest that the combination of olfactory and visual signals alter male zebrafish's mating-like behaviors via GnRH signaling.

## Introduction

The engagement of sexual behaviors is regulated by a number of factors, such as the expression of specific gene in sex-related cellular pathways, activation of hormone receptors, and stimulation of sensory cells [1–4]. Genetic loci that regulate the sexual behaviors have been identified. In flies (*Drosophila melanogaster*), for example, the expression of *fruitless* is required for initiating the courtship behaviors between the male and female animals; when the expression of *fruitless* is interrupted, the males alter their sexual behaviors [5,6]. Functional expression of *fruitless* in a variety of neural and non-neural tissues (e.g., olfactory bulb, visual cortex, auditory cells, and the gustatory system) is required for normal sexual behaviors [2, 7, 8].

Environmental cues such as pheromone modulation of sexual behaviors have been carefully examined [9, 10]. It is believed that in addition to pheromones, other sensory inputs, particularly those that trigger brain system GnRH signal transductions, may also have an impact on animal sexual behaviors [11, 12]. In vertebrates, different types of GnRH-containing cells have been identified [13–15]. Among them, the hypophyseotropic GnRH-containing neurons are found in all vertebrate species. These neurons are located in the pre-optic area and hypothalamus, and in some species, in the olfactory bulb [16, 17]. It has been speculated that the integration of cross-model sensory information may impact hypophyseotropic GnRH neural activity, which in turn, modulate animal’s sexual activity.

The zebrafish (*Danio rerio*) provide a model for studying the mechanisms underlying the integration of cross-model sensory information in sexual behaviors. In zebrafish, a paralogous form of mammalian hypophyseotropic GnRH-containing cells, known as the terminalis neurons (TNs) are found in the olfactory bulb [18–22]. Through the olfacto-visual centrifugal pathway, the TNs project axons to the neural retinas. In the retina, the TN axons synapse with dopaminergic interplexiform cells (DA-IPCs) and retinal ganglion cells (RGCs) [18, 23, 24]. While most of the RGC project axons to the visual cortex, some of the RGCs directly project axons to the hypothalamus [25]. This raises the possibility that through the olfacto-visual centrifugal pathway, the integration of multi-sensory information (i.e., between olfaction and vision) modulates neural functions in the hypothalamus, which in turn, alters animal’s sexual activity. In this research, using the zebrafish models we videotaped the sexual behaviors in adult zebrafish and recorded TN neural activities in transgenic animals under different experimental conditions. The data provide evidence that through the olfacto-visual centrifugal pathway, the integration of cross-model sensory information alters the dynamics of GnRH signaling in the brain and changes the sexual orientation in male animals.

## Materials and methods

### Animals

Zebrafish (*Danio rerio*) were maintained and handled in accordance with the NIH guidelines. All the experimental procedures were approved by the University of Notre Dame IACUC. Adult zebrafish were kept in 5-liter tanks (30 fish per tank, approximately half males and half females) or otherwise specified. The fish were maintained in the normal light-dark cycle (14:10; light, 07:00–21:00) and fed two times a day with freshly hatched brine shrimps.

### Score of sexuality

Zebrafish were videotaped during their normal mating times (between 07:00 and 07:15). In the evening before the recording was taken, male and/or female zebrafish were transferred to the mating container (8 inches x 3 inches x 3 inches, long x wide x deep). The container was divided into two parts by an aluminum screen that was inserted in the middle of the container. The screen permitted water flow but separated the fish from physically contacting with each other. Each half of the container was further divided into 4 rectangle sections by lines marked on a paper that was placed underneath the container. The lines could be visualized through the bottom of the transparent container. Animal behaviors were videotaped and the traces of swimming behaviors were plotted in 15-sec intervals.

Several measures were put in place to indicate the animal’s sexuality levels. First, we recorded the speed of their swimming and scored each category of swimming behaviors: slow swimming or stationary was given a score of 0, normal swimming was given a score of 1, fast swimming was given a score of 3, and swimming against the net was given a score of 5. Data were collected in 3-min periods, and total 5 measurements were performed during the entire 15-min videotaping period. For each 3-min recording period, the percentage of time of each type of swimming was calculated, scaled down by a factor of 10, and then multiplied by the scores obtained from their swimming behaviors. Second, we examined the locations of the fish (at 15-second intervals) in the container in relation to the net. If the fish was recorded in the half of its side close to the net, it scored 5 points, and if the fish swam in the half of the side further from the net, it scored 0 points. Total scores from swimming speeds and locations were summated to indicate the sexuality levels. For the purpose of comparison, the scores from the most sexually active control male fish was normalized to 1, and the sexuality levels of other animals were determined by comparing to the base value of 1.

### Electrophysiological recordings

Single-unit TN recordings were performed in developing transgenic zebrafish Tg(GnRH-3::GFP) between 4 and 5 days old. Prior to recordings, the fish were anesthetized with 0.5% 3-amino benzoic methylester and then embedded in 1% low-melting agrose. Patch pipettes (7–10 MΩ were fabricated from borosilicate glass (Sutter Instrument Co, CA) and filled with the pipette solutions (150 mM NaCl and 10 mM HEPES, pH 7.4). Whole-cell recording configurations were obtained by a brief pulse of suction after a gigaohm (GΩ) seal. For loose-patch recordings, once a spike was detected, light suction was applied to establish a low-resistance seal (50–100 MΩ). Voltages were recorded in the current-clamp mode using Clampex 8.0 software connected to an Axopatch 200B amplifier and a Digidata 1322A digitizer (Axon Instruments, CA). For each set of recording, data were collected in 60-second periods.

Drug solutions [glutamate, 6-cyano-7- nitroquinoxaline-2,3-dione disodium salt (CNQX; 10 μmol/L), D(–)-2-amino-7-phosphonoheptanoic acid (D-AP-7; 100 μmol/L); Sigma, MO] were delivered to the olfactory bulb (adjacent to the TNs, which were revealed by the expression of the GFP) at a rate of 10 μl/s.

### Odor stimulation

Amino acids (methionine) were used for odor stimulation. Stock solutions (50 mM) were freshly prepared each day for individual experiment. Methionine (Sigma, MO) were dissolved in distilled water, and kept in room temperature. Methionine were delivered to swimming water (to the center of the container for adult zebrafish behavioral test) or perfused to the nostril using a glass pipette (for TN recordings) at the final concentration of 1.2 mM.

### Chemical lesion

Olfactory neurons were ablated using 0.7% Triton X-100 [26]. Prior to drug treatment, the fish were anesthetized in a small container by 0.02% 3-aminobenzoic acid ethyl ester (applied to swimming water). Under a dissecting microscope, 0.5μl of 0.7% Triton X-100 (in PBS) were delivered to the nostril of the fish. Both sides of the nostril were treated. Control treatment was conducted using PBS. The fish were allowed to recover 2 days before used for mating tests.

DA-IPCs and RGCs were ablated by intraocular injections of 6-OHDA (5 μg/μl) and ouabian (3 μM), respectively. The fish were anesthetized by 0.02% 3-aminobenzoic acid ethyl ester before receiving injections. Under a dissecting microscope, 1 μl of drug solutions were injected into the vitreous. The fish were allowed to recover 4 days before used for mating tests.

### Statistical analysis

Different numbers of zebrafish were used for different sets of the experiment. For the behavioral tests, under each condition (e.g., sham or methionine stimulation, control or lesion experiment) and for each mating-set (e.g., male-female, male-male, female-females), 6 pairs of zebrafish (between 4 and 6 months old) were tested. For electrophysiological studies, under each test condition (control, glutamate stimulation with or without receptor blockers, or in response to sham or methionine stimulation), 6 GFP-tagged TN cells from different transgenic animals (between 4 and 5 days old) were recorded. Data were analyzed using the double-blind methods. Statistical differences between the control and experimental or between sham and drug treatment groups were determined using the Student t-test.

## Results

### Activation of the olfacto-visual centrifugal pathway alters male zebrafish’s sexual orientation

**Fig 1:**
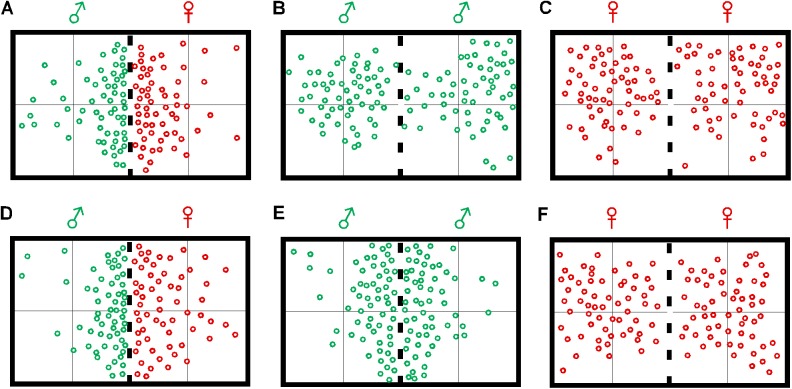
Static positions of locomotor behaviors of male and female zebrafish recorded in control (**A-C**) and odor-stimulation conditions (**D-F**). The fish were separated by a net inserted in the middle of the container (indicated by bold dashed lines). Data were plotted in 15-sec intervals for a total of 15 min. Under control conditions (no odor stimulations), in pair-wise male-female setting, both the male and female fish show high sexuality levels, i.e., they displayed fast swimming behaviors and spent most of the time swimming close to the net in attempt to contact each other. In male-male or female-female settings, the fish randomly swam in the container, and they did not spend extra time swimming near the net. In response to odor stimulation, the behavior of the fish in the male-female setting remained unchanged. However, in the male-male setting, upon odor stimulation, the fish showed high sexuality levels. They increased the speed of swimming, and spent most of the time swimming close to the net in attempt to interact with each other. No obvious changes in swimming behaviors were seen in the female-female setting.

We videotaped the behaviors of adult male and female zebrafish during their normal mating times in the early morning. In the control setting (1 male and 1 female in the same container but separated by a net), both the male and female zebrafish displayed behavioral characteristic of mating. For example, for much of the time during the 15-min recording period, the male and female zebrafish swam close to the net, and often, bumped into the net in an attempt to interact with each other (Fig 1A). This was observed in 5 out 6 pairs tested on different days. In settings that two males (n = 6) or two females (n = 6) were kept in the same container and separated by a net, no obvious mating-like behaviors were observed. At most times during the 15-min recording period, the fish slowly swam around, and sometimes remained still. They did not spend much time swimming near the net (Fig 1B and 1C).

Animal sexual behaviors are regulated by internal circulation of GnRH [27]. In zebrafish, a group of GnRH-containing neurons (the TNs) are found in the olfactory bulb [18, 28]. The TNs express glutamate receptors and can be activated by glutamate or glutamate receptor agonists, such as NMDA or KA [23]. Because the TNs synapse with the RGCs [18], and because some of the RGCs directly project axons to the hypothalamus [25], it is possible that through the olfacto-visual centrifugal pathway, cross-model sensory information integration (i.e., between the olfactory bulb and retina) may alter the dynamics of GnRH signaling in the brain, which then impact the animal’s sexuality. We characterized the sexual behaviors of zebrafish in response to odor stimulation. Methionine was chosen as the odor stimulant [28, 29]. In the control setting (1 male and 1 female in the same container and they were separated by a net), activation of the olfacto-visual centrifugal pathway (by 1.2 mM methionine, applied to swimming water) produced no obvious effect on animals’ mating behaviors. For much of the time during the 15-min recording period, both the male and female fish swam close to the net in attempt to interact with each other (Fig 1D).

When two males were kept in the same container and separated by a net, stimulation by methionine led to increased mating-like behaviors. For example, both of the males showed increases in fast swimming behaviors, and they spent most of the time swimming close to the net. Often, they swam into the net in an attempt to make contact with each other (Fig 1E). However, when two females were kept in the same container and separated by a net, activation of the olfacto-visual centrifugal pathway by methionine produced no effect on the animal’s sexual behaviors, i.e., both fish swam slowly and did not appear to be interested in interacting with each other (Fig 1F).

### Stimulation of olfactory neurons increases the activity of the TNs

**Fig 2:**
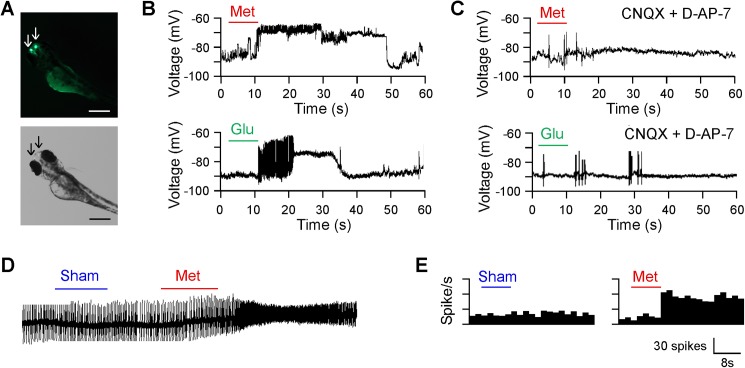
Effects of odor or glutamate stimulation on TN activity. **(A)** Fluorescent and bright-field images of transgenic zebrafish Tg(GnRH3::GFP). The TNs were identified by the expression of GFP (arrows) and were recorded by patch-clamp methods. Scale bar: 200 μm. **(B)** Whole-cell patch-clamp recordings of the TN in response to odor (methionine, 1.2 mM applied to the nostril) or glutamate (0.5 mM, applied to the olfactory bulb) stimulation. Note the increase in membrane potential of the TNs in response to odor or glutamate stimulation. In all cases (n = 6), the holding potential was set at -90 mV. **(C)** Whole-cell patch-clamp recordings of the TNs in response to odor (methionine, 1.2 mM applied to the nostril) or glutamate (0.5 mM, applied to the olfactory bulb) stimulation in zebrafish in which the glutamate receptors were blocked by CNQX or D-AP-7. In response to odor or glutamate stimulation, no obvious changes were detected. In all cases (n = 6), the holding potential was set at -90 mV. **(D)** Effects of odor stimulation (methionine, 1.2 mM applied to the nostril) on spontaneous firing of the TN neuron. Note the increase of the firing in response to odor stimulation. Sham stimulation (with distilled water) produced no effect on the firing rate of the TN neuron. The experiments were performed using different TN neurons (n = 6). **(E)** Histograms of the firing of the TNs (n = 6) in response to sham (distilled water) or odor stimulation (methionine, 1.2 mM; applied to the nostril). Note the increase of the firing in response to odor stimulation.

To test if the changes of the animal’s sexual behaviors after odor stimulation may relate to olfacto-visual centrifugal GnRH signaling, i.e., if the TN cells respond to increases in glutamate concentrations after odor stimulation, we recorded the TNs in live animals in response to odor stimulation using transgenic zebrafish Tg(GnRH3::GFP). In developing transgenic fish, the GFP-tagged TNs can be readily identified and manipulated (Fig 2A) [23, 30]. Stimulation of the olfactory neurons (by 1.2 mM methionine, applied to the nostril) produced long-lasting effects on the TNs, such as increases in membrane potentials (Fig 2B). Directly applying glutamate (0.5 mM, applied to the olfactory bulb) mimicked the effect of odor-mediated increase of membrane potential of the TNs (Fig 2B). In zebrafish that were treated with glutamate receptor antagonists CNQX or D-AP-7 (0.05 mM, applied to the olfactory bulb prior to odor stimulation to the nostril or glutamate stimulation to the TNs), the membrane potential of the TNs remained unchanged in response to olfactory odor or glutamate stimulations (Fig 2C).

Loose-patch recordings of the TNs revealed that stimulation of olfactory neurons (with 1.2 mM methionine, applied to the nostril) increased the firing rate of the TNs (Fig 2D). As compared to the firing rete recorded prior to odor stimulation, on average the firing rate of the TNs increased 140.55 ±27.82% in response to odor stimulation (Fig 2E).

### Interruption of the olfacto-visual pathway diminished mating-like behaviors between males

**Fig 3:**
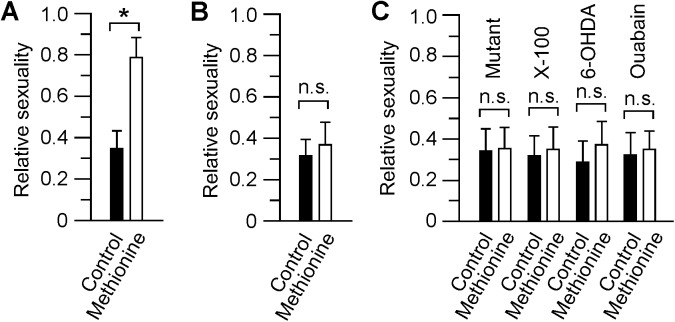
Relative sexuality levels of wild-type, mutant, and chemically lesioned animals tested under different conditions. **(A)** Sexuality levels of male zebrafish recorded in the male-male setting before (black bar) and after odor stimulation (white bar). Note the increase of sexuality levels after odor stimulation. (**B)** Sexuality levels of female zebrafish recorded in the female-female setting before (black bar) and after odor stimulation (white bar). No obvious changes were observed before and after odor stimulation. (**C**) Sexuality levels of nbb and drug-treated males (Triton X-100, which destroyed the olfactory neurons; 6-OHDA, which ablated DA-IPCs; ouabain, which selectively lesioned RGCs) in the male-male setting recorded before (black bar) and after odor stimulation (white bar). In all cases, no obvious changes in sexuality levels were detected after odor stimulation. The data represent the Means ± SE. n = 8 in each group. * p < 0.05; n.s., not significant.

To further investigate the role of olfacto-visual sensory integration in male zebrafish’s sexual behaviors, we measured the sexuality levels of males that were placed in the same container without separation by the net. The experiments were conducted in wild-type fish and in fish with interruption of the olfacto-visual centrifugal pathway due to gene mutation or chemical lesion. In control wild-type fish, when two males were put together, for much of the time during the 15-min recording period both fish slowly swam around in the container and showed no interactions. Activation of the olfacto-visual centrifugal pathway (by 1.2 mM methionine, applied to swimming water) triggered mating-like behaviors. For example, upon the application of methionine, both males became occupied by chasing each other around the container, i.e., one male followed the other male by aligning its head next to the side of the other male slightly below the anal fin [31–33]. Based on their locomotory patterns (e.g., swimming speed) and mutual interactions (e.g., chasing behaviors), we scored the sexuality level of the fish. In males, activation of the olfacto-retinal centrifugal pathway increased the sexuality level by approximately 2-fold as compared to those measured under control conditions (Fig 3A). Interestingly, odor-triggered increases in sexuality were only observed in male animals. In wild-type females (the fish were kept in the same container without separation by the net), the fish showed no changes in sexual behaviors and the sexuality levels remained low with or without stimulation by odors (Fig 3B).

In zebrafish *night blindness b* (*nbb*) mutants, the olfacto-visual centrifugal pathway is interrupted, for example, the number of TN axons that project to the retina is reduced, the pattern of TN fiber distribution in the retina is disorganized, and the number of DA-IPCs is decreased [18]. In normal pair-wise setting, the sexual behaviors of *nbb* males were indistinguishable from wild-type animals, i.e., they mated with females and produced progenies. When two *nbb* males were placed together, they showed no mating-like behaviors either before or after the application of odors (1.2 mM methionine, applied to swimming water) (Fig 3C). In wild-type males in which the olfacto-visual centrifugal pathway is blocked (i.e., by treatment with Triton X-100, which ablate the olfactory neurons) [26], or the targets of the olfacto-retinal centrifugal pathway (i.e., DA-IPCs or RGCs in the retina) were excised by 6-OHDA or ocubain [34, 35], under normal pair-wise conditions, their mating behaviors were indistinguishable from wild-type males. However, when two chemically-treated males were kept together, in response to odor stimulation (1.2 mM methionine, applied to swimming water), during the full 15-min recording period, their sexuality levels remained low, similar to those measured before the application of methionine (Fig 3C).

## Discussion

It is known that the circulation of GnRH is regulated by different genetic and cellular events [36]. However, the effect of environmental input on the regulation of synthesis and release of GnRH and how external modulation of GnRH signaling transduction impacts the sexual behaviors remain to be examined. Zebrafish provide a model system for addressing these issues. Zebrafish possess two distinctive forms of GnRH, which include cGnRH and sGnRH [37–41]. Previous studies have shown that the increase of cGnRH signaling promotes animals’ survival functions, such as food intake, whereas the modulation of sGnRH signaling transduction directly affects animals’ sexual activity and reproduction. In zebrafish, the olfactory TNs synthesize and release sGnRH [19]. Previously, we demonstrated that through the olfacto-visual centrifugal pathway, activation of the olfactory neurons decreases the release of dopamine from retinal DA-IPCs. This leads to two consequences: First, it increases the coupling between horizontal cells in the outer retina [42, 43]. Second, it lifts the inhibition of dopaminergic signaling on RGCs, thereby increasing inner retinal activity [44]. Together, the integration of olfacto-visual sensory information increases visual perception and retinal sensitivity. The increase of visual perception and retinal sensitivity directly impacts the animal’s social behaviors, which include sexual mating [45, 46].

**Fig 4:**
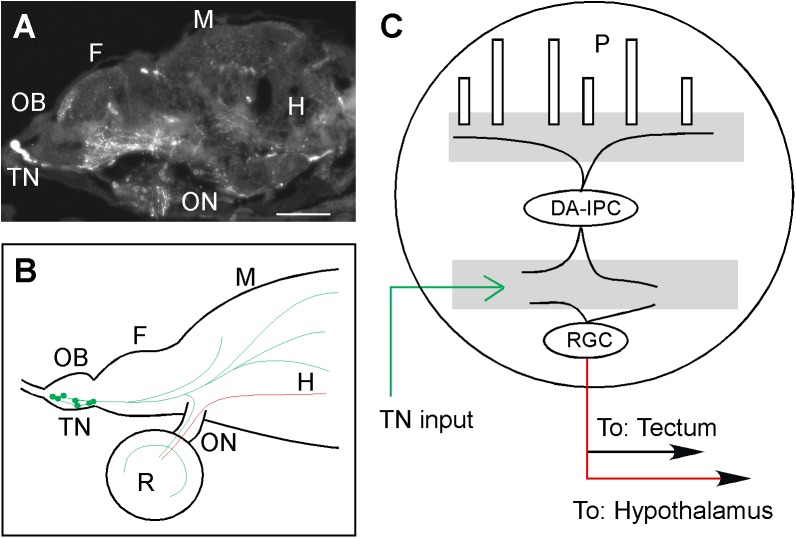
Effects of GnRH-mediated olfacto-visual sensory integration on animal sexuality. **(A)** Lateral view of transgenic zebrafish (GnRH::3-GFP; anterior to the left) that shows the TNs and TN axons in the brain (revealed by the expression of the GFP). Scale bar, 50 μm. **(B)** A cartoon showing the projection of TN axons (red) and the projection of RGC axons (green) to the hypothalamus. **(C)** GnRH signaling in the eye. Upon entering the retina, the TN axons synapse with DA-IPCs and RGCs. Most of the RGCs project axons to the visual cortex, but some of the RGCs project axons to the hypothalamus. Abbreviations: F, forebrain; H, hypothalamus; M, midbrain; P, photo receptor cells; R, retina; DA-IPC, dopaminergic interplexiform cell; INL, inner nuclear layer; IPL, inner plexiform layer; OB, olfactory bulb; ON, optic nerve; OPL, outer plexiform layer; RGC, retinal ganglion cells; TN, terminalis neuron.

Activation of the olfactory neurons increases the firing rate of the TNs, suggesting that through the olfacto-visual centrifugal pathway, sensory inputs alter the dynamics of GnRH signaling in the brain. The increase of TNs firing after odor stimulation is mediated by activating the glutamate receptors expressed on the TNs. When the glutamate receptors are blocked by receptor antagonists, stimulation of the olfactory neurons produces no effect on TN activity. The data suggest that through GnRH signaling, the olfacto-visual pathway may function as a bridge that incorporates external sensory signals to the sex-brain (see Fig 4). It works through the following mechanisms: Stimulation of olfactory neurons increases glutamate release in the olfactory bulb; since the TNs express glutamate receptors, thus odor stimulation will activate the TNs. Upon the activation of the TNs, internal GnRH-signaling will be increased. Through the olfacto-visual centrifugal pathway, the increase of GnRH signaling will be propagated to the retina. Because the RGCs express GnRH receptors [47], it is possible that the increase of GnRH signaling due to odor stimulation will alter the firing patterns of the RGCs. Because some of the RGCs project axons to the hypothalamus; the increase of GnRH signaling triggered by odor stimulation will then alter the activity of hypothalamus neurons. Alterations in GnRH signaling in the brain will directly impact the animal’s sexual activity.

Our data provide evidence for the involvement and the physiological relevance of multi-sensory information integration in modulation of animal sexual activity. This is observed only in males. In pair-wise male-female or female-female settings, the activation of the olfacto-visual centrifugal pathway produced no obvious effect on animal sexual behaviors. It is possible that the GnRH signaling propagated by the olfacto-visual centrifugal pathway only targets male-specific dimorphic cells [6, 48, 49]. When the male fish encounter the other fish (regardless it is a male or a female), the male shows mating-like chasing behaviors.

Previous studies have demonstrated that in some of the mammalian species (e.g., rams), differences in brain structures due to the exposure to sex-related hormones, such as testosterone, may be involved in alterations in sex-orientations in male animals [50]. The increase of sexual activity induced by male-specific hormones, however, can be inhibited by chronic treatment with GnRH or its agonists [51]. In addition, sensory inputs, such as those mediated by the olfactory and/or vomeronasal systems, also play important roles in males’ sex behaviors [52]. The underlying mechanisms remain to be investigated.
